# Diagnosis and management of refractory and rare moulds

**DOI:** 10.1093/jac/dkaf031

**Published:** 2025-03-14

**Authors:** Jame Hurley, Esteban Martinez

**Affiliations:** Melbourne Medical School, The University of Melbourne, Melbourne, Australia; Infectious Diseases Unit, Hospital Clinic and University of Barcelona, Barcelona 08036, Spain

To prescribe antifungals appropriately requires access to information and guidance from multiple sources. This is especially challenging given the rise in invasive fungal infections and disease and the availability of newer antifungal agents over the past two decades.

This supplement includes four articles that were derived from a symposium of the European Conference on Clinical Microbiology and Infectious Diseases conference in 2023.

In their article on Coccidioidomycosis, Drs Donovan, Fernandez, Bains and di Pompo deal with aspects of the epidemiology, clinical presentations, diagnosis, treatment and prevention of this disease. Coccidioidomycosis represents a growing concern in relation to an expanding geographical range with possible clinical and economic consequences beyond the USA. There is hope that newer therapies will emerge from clinical trials for this difficult-to-treat infection.

The diagnosis and management of invasive fungal infections due to non-*Aspergillus* moulds is discussed by Dr Morrissey. These infections are a significant cause of morbidity and associated with high mortality rates. She discusses emerging diagnostic and therapeutic strategies. These are coming to attention increasingly due to the number of immunosuppressed patients in our populations. There is some hope that modulation of the immunosuppression may be useful as a strategy.

The management of invasive aspergillosis is difficult at the best of times but the management of refractory and resistant disease is especially challenging. Professors Arendrup and Cordonnier address the issues involved here including what constitutes refractory disease and how to define resistance for this rare disease. They discuss how the emergence of newer therapies will create new opportunities for this condition.

In the first article, Professor Pagano and Dr Fernández discuss the clinical aspects and recent advances in fungal diseases impacting human health including specific challenges relating to awareness, diagnosis, limited treatment options, the occurrence in immunosuppressed individuals and the possible environmental origins of these infections.

These articles provide support for appropriate antifungal use including the newer agents, an overview, and recommendations for strategies to respond to the rise in invasive fungal infections and disease.

We thank the authors wholeheartedly for their contributions. We hope that readers find this special issue as interesting, educational and enjoyable to read as it has been for us to edit!

